# Real-world experience with anti–programmed cell death protein 1 immunotherapy in patients with esophageal cancer: A retrospective single-center study

**DOI:** 10.3389/fonc.2022.880053

**Published:** 2022-09-09

**Authors:** Xinpeng Wang, Lvjuan Cai, Mengjing Wu, Guo Li, Yunyun Zhu, Xinyue Lin, Xue Yan, Peng Mo, Huachun Luo, Zhichao Fu

**Affiliations:** ^1^ Department of Radiotherapy, The 900th Hospital of the Joint Logistics Team, Fujian Medical University, Fuzhou, China; ^2^ Department of Radiotherapy, Dongfang Hospital of Xiamen University, School of Medicine, Xiamen University, Xiamen, China

**Keywords:** esophageal cancer, immunotherapy, retrospective study, efficiency, safety

## Abstract

The “real-world” data of programmed cell death protein 1 (PD-1) inhibitors in esophageal cancer (EPC) are still an unmet medical need, including the clinical efficacy and safety. Seventy-seven EPC data were studied retrospectively; the progression-free survival (PFS), risk factors (clinical stages larger than stage II, metastatic sites larger than 2, treatment lines larger than the first line, previous surgical treatment, combined positive score [CPS] expression, etc.), and the safety were analyzed. The median PFS for all patients was 7.2 months, clinical stage > stage II; the number of treatment lines > first line was significantly correlated with prognosis (all *P* < 0.05). Subgroup analysis showed that the median PFS of patients with clinical stage ≤ II was better; the results were the same for the patients with ≤2 metastatic sites, first-line PD-1 inhibitors, and not previously received radical surgery (all *P* < 0.05). Meanwhile, the incidence of adverse events (AEs) of varying degrees was 25.97% (20/77) in 20 patients and 6.49% (5/77) of grade 3/4 AEs. The highest AE was myelosuppression (15.58%), followed by liver function injury (7.79%). In addition, ≥2 lines of treatment and >2 metastatic sites predicted poor outcomes for patients with EPC who had failed first-line therapy or progressed with the combined immunotherapy and chemotherapy treatment strategy (all *P* < 0.05).

## Introduction

According to the data of GLOBOCAN 2020, of the 4,568,754 new cancer cases in China in 2020, 38.8% are malignant tumors of the digestive tract, of which the age-standardized incidence rate (ASIR) of esophageal cancer (EPC) ranks the 6^th^ place among all tumors (13.8 cases per 100,000 people). Of the 3,002,899 patients with new cancer-related deaths, the age-standardized death rate (AMSR) of EPC ranked the 4^th^ (12.70 per 100,000 people) (1). EPC has high morbidity and mortality. Although good progress has been made in the traditional treatment model or combination of surgery, radiotherapy, and chemotherapy (2), it is still difficult to meet the expectations of further improving the prognosis of patients.

The emergence of immune checkpoint inhibitors (ICIs) broke this situation. For any patient who responds to the treatment of multiple cancers, ICIs can provide long-term disease control and significantly improve the survival rate and the quality of life of patients (3). The Checkmate-577 study opened a chapter in adjuvant immunotherapy for EPC, suggesting that immunotherapy can significantly improve patients’ DFS (4). The studies on ATTRCTION-3 (5), ESCORT (6), and KEYNOTE-181 (7) have shown that immunotherapy, as a treatment for advanced second-line EPC, has significantly better Objective Response Rate (ORR) and Overall Survival rate (OS) than the chemotherapy control group and has good safety. The subgroup analysis data of the KEYNOTE-181 (7) study showed that Asian populations may have more survival benefits from immunotherapy. The data of the interim study of KEYNOTE 590 (8) and ESCORT-1^st^ released at the 2020 ESMO conference suggested that, in the advanced first-line treatment, the OS and progression-free survival (PFS) of the immunotherapy combined with chemotherapy group were significantly better than those of the control group. NCT02743494, CheckMate 648, and Checkmate-649 are expected to further prove that immune-combined chemotherapy and dual immunotherapy have significant benefits in OS and ORR, regardless of PDL1 expression (9). In addition, RATIONALE205 for patients with locally advanced EPC shows that ICI combined with chemotherapy has good efficacy and safety. Studies on KEYNOTE-975, SHR-1210-III-323, and RATIONALE311 (10) further explored the role of concurrent chemoradiotherapy combined with ICI therapy in locally advanced EPC.

More and more evidence supports that immunotherapy and immune-based combined therapy can significantly improve the prognosis of patients with EPC. However, the efficacy and safety of ICI treatment should still be further verified in the real world. Therefore, we retrospectively analyzed the esophagus patients treated in our center for immunotherapy in the past 3 years, evaluated their real safety and efficacy, and further determined relevant factors that significantly affected their prognosis.

## Materials and methods

### Patients

**Figure 1:**
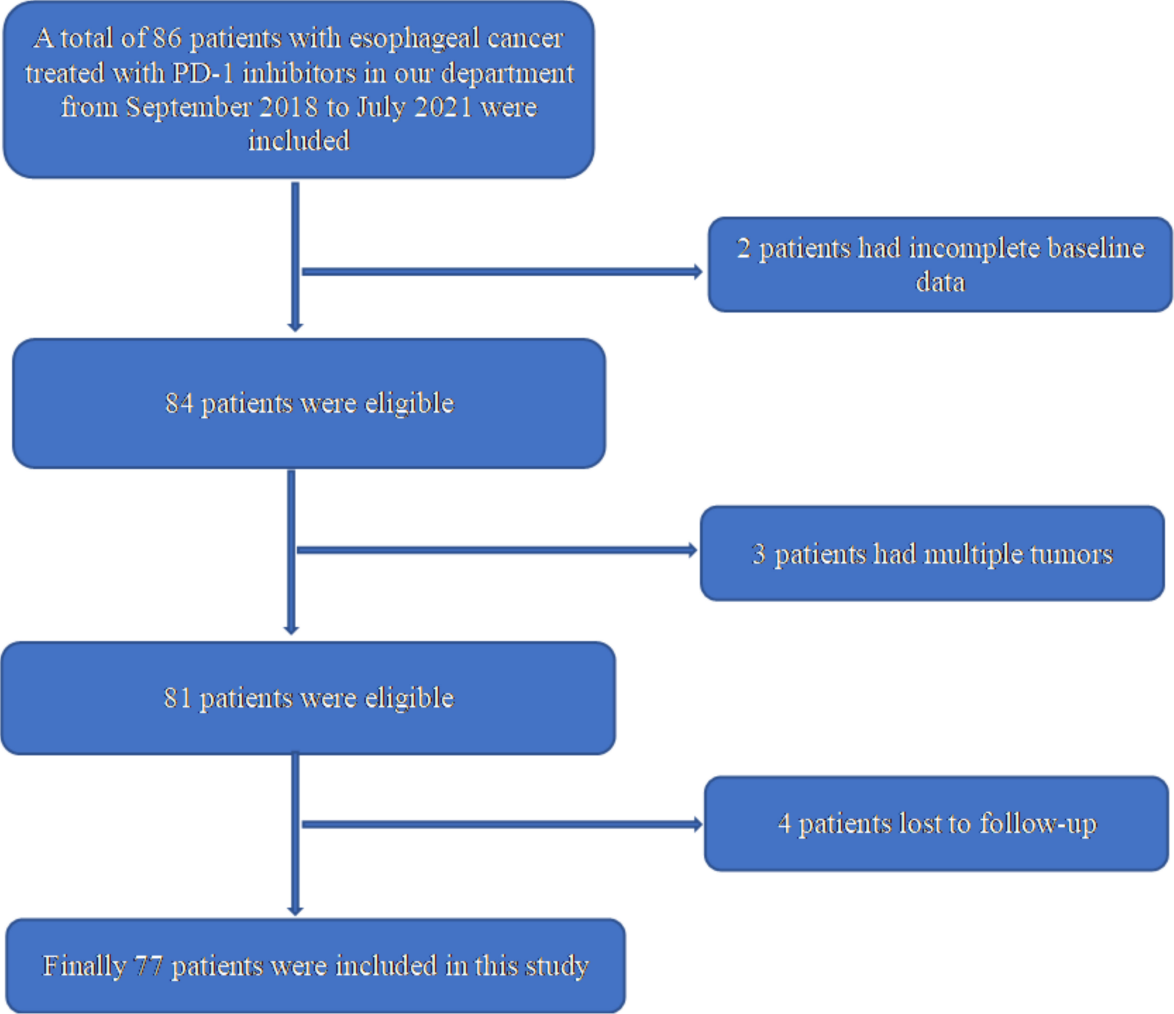
Patient selection process for the retrospective cohort.

This retrospective study included a total of 86 patients with EPC who had received ICI treatment in the Radiotherapy Department of the 900th Hospital of the Joint Logistics Support Force between September 2018 and July 2021. Inclusion criteria: (1) age ≥ 18 years; (2) diagnosis of EPC by pathological histology; (3) presence of at least one measurable lesion prior to treatment according to RECIST 1.1 tumor evaluation criteria; (4) patients with no co-infections or other serious systemic diseases before treatment. Exclusion criteria: (1) combination of other malignancies, except cured basal cell carcinoma of the skin or squamous carcinoma of the skin or any other *in situ* carcinoma; (2) presence of any abnormal bone marrow hyperplasia and other hematopoietic disorders prior to treatment; (3) those with active infection requiring treatment, HIV infection, viral hepatitis before treatment; (4) Patients with other serious systemic diseases require pharmacological intervention. Of them, nine patients were excluded: Two patients were excluded due to incompleteness of baseline data, three patients due to having multiple tumors, and four patients due to being lost to follow-up. Finally, 77 patients were included in this study (**Figure 1**). All patients in the group met the pathological diagnostic criteria for EPC, including one case of adenocarcinoma, one case of neuroendocrine cancer, and the rest cases were esophageal squamous cell carcinoma. The study data were collected through patient electronic medical records and telephone follow-up. The clinical data were collected and analyzed retrospectively, including baseline clinical characteristics of patients, PDL-1 expression, disease progression and the time of death of patients, and treatment-related adverse events (AEs). All patients were informed and accepted the treatment protocol during the previous treatments.

### Treatment

ICI includes pembrolizumab, toripalimab, camrelizumab, sintilizumab, and tilelizumab. The doses of pembrolizumab, carrelizumab, sintilizumab, and tilelizumab received by the patients were fixed doses of 200 mg every 3 weeks. The therapeutic dose of toripalimab is a fixed dose of 240 mg every 3 weeks.

The chemotherapy regimen includes adjuvant chemotherapy regimen: cisplatin combined with fluorouracil: cisplatin 60–80 mg/m^2^, i.v., d1; fluorouracil 1,000 mg/m^2^•d, i.v., d1-5. It is repeated every 3 weeks. Paclitaxel combined with cis-platinum: paclitaxel 150–175mg/m^2^, i.v., d1 or 80 mg/m^2^, i.v., d1, 8; cisplatin 60–75mg/m^2^, d1 or d2. It is repeated every 21 days. Docetaxel combined with platinum: docetaxel 60–75 mg/m^2^, i.v., d1 or 30–35 mg/m^2^, i.v., d1-2; cisplatin 70 mg/m^2^, i.v., d1 or nedaplatin 50 mg/m^2^, i.v., d1. It is repeated every 3 weeks. Capecitabine combined with paclitaxel: capecitabine 1,000 mg/m^2^, p.o., bid, d1-14; paclitaxel 80 mg/m^2^, i.v. d1, 8. It is repeated every 3 weeks. Capecitabine combined with cisplatin: capecitabine 825–1,000 mg/m^2^, p.o, bid. d1-14; cisplatin 75 mg/m^2^, i.v., d1. It is repeated every 3 weeks. Capecitabine combined with docetaxel: capecitabine 825–1000 mg/m^2^, p.o, bid, d1-14, an interval of 7 days; docetaxel 60 mg/m^2^, i.v., d1. It is repeated every 3 weeks. Paclitaxel single agent: paclitaxel 60–80 mg/m^2^, i.v., d1, 8, 15, and it is repeated every 4 weeks. Docetaxel monotherapy: docetaxel 60–75 mg/m^2^, i.v., d1, and it is repeated every 3 weeks. In view of toxic and side effects of combined therapy, no combination of three or more chemotherapy drugs has been used.

Concurrent chemotherapy will include a combination of cisplatin (25 mg/m^2^, IV; days 1–3 of each 3-week cycle) and paclitaxel (135 mg/m^2^, i.v.; day 1 of each 3-week cycle), and two cycles will be given. Adjuvant or palliative chemotherapy regimens include FP regimen (5-FU 800 mg/m^2^ d1-5 Q3W + cisplatin 80 mg/m^2^ Q3W) and TP regimen (albumin paclitaxel 260 mg/m^2^ i.v. + cisplatin 75 mg/m^2^).

The radiotherapy regimen includes radical concurrent chemoradiotherapy with a dose of 50–60 Gy, and postoperative adjuvant radiotherapy with a dose of 45–50.4 Gy (all included patients are R0 resection), and concurrent chemoradiotherapy will be divided into 28 sessions (total dose: 50.4 Gy).

Surgical treatment method: thoracoscopic radical resection of EPC + regional lymph node dissection. The start of the operation is after the confirmation by clinical or biopsy pathological diagnosis, or 6–8 weeks after the end of neoadjuvant chemoradiotherapy, or 3–6 weeks after the end of neoadjuvant chemotherapy.

We used immunotherapy in combination with chemotherapy for stage II (three patients), stage III (nine patients), stage IVA (two patients), stage IVB (20 patients) who received second-line treatment after progression, and for stage IVB patients (12 patients) who received first-line treatment. Two of the stage IVB patients were treated with a combination of anti-vascular targeting agents. Of the remaining patients, six stage I patients underwent surgery and received postoperative immune maintenance therapy. Neoadjuvant immunotherapy in conjunction with chemotherapy was given to four stage II patients and five stage III patients. Induction immunotherapy was given to a further two stage II patients and five stage III patients, along with radical concomitant chemoradiotherapy and immunotherapy. Neoadjuvant chemotherapy and postoperative immune maintenance treatment were used to treat two stage III patients. Postoperative adjuvant immunotherapy combined with chemotherapy was used to treat the remaining stage III patients. Following radical radiation, immune maintenance treatment was given to the remaining three-stage IVA patients.

### Ethics statement

The studies involving human participants were reviewed and approved by the ethical review board committee of the 900th Hospital of the Joint Logistics Team. The patients provide their written informed consent to participate in this study.

### Evaluation

Tumor staging was based on the 2017 TNM staging standard of the American Joint Committee on Cancer (AJCC). Efficacy was assessed according to RECIST 1.1 tumor evaluation criteria. PFS was defined as the time from the baseline evaluation level of treatment before ICI was used to the imaging (enhanced CT), suggesting the progression of the disease. OS was defined as the time from the baseline evaluation level of treatment before ICI use to death from any cause. CTCAE version 5.0 was used as the standard to evaluate the grade of AEs during the treatment of patients. Statistical criteria for PD-L1 expression: TPS was defined as the percentage of tumor cells stained with PD-L1 membranes at any intensity. CPS was defined as the sum of the number of PDL1-stained tumor cells and tumor-associated immunity per 100 tumor cells.

### Statistical analysis

The patient’s PFS was estimated using the Kaplan–Meier method. The 95% CI was calculated using the Brookmeyer–Crowley method. The univariate stratified comparison was made through the log-rank test. Then, the covariate (*P* < 0.05 in the univariate analysis) was entered into the regression model of multivariate Cox proportional hazards. The HR for PFS and corresponding 95% CI were calculated with the Cox proportional hazards model. A forest map was plotted for further comparison and analysis. Statistical analysis was performed using SPSS software (version 26.0, IBM Software, Armonk, NY, USA) and R software (version 4.1.0).

## Results

### Clinical characteristics and treatment

The retrospective study cohort included 77 patients with EPC who had previously received ICI treatment. The median follow-up time was 7.9 ± 1.867 months by the time the data were locked (30 December 2021).

The median age was 60 years (45–74 years), and 37 patients (48.1%) were over 60 years old. Of the 77 patients, 60 (77.9%) were men and 17 (22.1%) were women. The ECOG performance status score (PS) of all included patients was lower than 2 points. Of the 77 patients, according to the staging standard of the 8^th^ edition of the AJCC, T3 patients accounted for the largest proportion (49.3%) of the T stage, and in the lymph node staging, the proportion of N1 and N2 patients accounted for the vast majority, respectively, 33 (42.8%) and 28 (36.4%). The included patients were mainly stages III and VI, 25 (32.5%) and 37 (48.0%), respectively, and six (7.8%) and nine (11.7%) patients were in stages I and II, respectively. Of all stage VI patients, 32 patients were stage VIB, in which 15 had metastases in a single organ, five patients had metastases in two positions, and the rest 12 patients had metastases in more than two organs. Among all the target organs for which metastasis has been observed, lung metastasis has the highest incidence (16 cases), followed by liver metastasis (12 cases), and the rest are bone metastasis (seven cases) and brain metastasis (two cases). Of the 77 patients, 43 (55.8%) patients received neoadjuvant, adjuvant, or first-line immunotherapy, and the rest 34 patients (44.2%) received ICI treatment after the second-line treatment. Thirty-six patients had previously undergone radical EPC ± lymph node dissection; 45 patients received radiotherapy, including preoperative neoadjuvant, postoperative adjuvant, and radical concurrent chemoradiotherapy, and 60 patients joined chemotherapy regimen before or during ICI treatment (including albumin paclitaxel or Tegafur, Gimeracil and Oteracil Potassium monotherapy regimen, and albumin paclitaxel plus platinum or Tegafur, Gimeracil, and Oteracil Potassium plus platinum combined regimen); two patients of second-line treatment were treated with anti-vascular–targeted therapy (apatinib and anlotinib, respectively).

In this study, there are five programmed cell death protein 1 (PD-1) checkpoint inhibitors available: sintilizumab (23.4%), carrelizumab (54.5%), teriprizumab (9.1%), tislelizumab (6.5%), and pembrolizumab (6.5%). According to the PDL1 expression of patients before treatment, 14 patients had PD-L1 CPS lower than 10%, 13 patients had PD-L1 CPS higher than or equal to 10%, and the PD-L1 expression levels of another 50 patients before treatment were not recorded (**Table 1**).

**Table 1:** Baseline characteristics.

Variables	Number of cases (%)
**Age (years)**	
Median (range)	60 (45-74)
< 60	37 (48.1)
≥ 60	40 (51.9)
**Gender**	
Male	60 (77.9)
Female	17 (22.1)
**T stage**	
1	4 (5.2)
2	22(28.6)
3	38 (49.3)
4	13 (16.9)
**N stage**	
0	13 (16.9)
1	33 (42.8)
2	28 (36.4)
3	3 (3.9)
**M stage**	
0	46 (59.7)
1	31 (40.3)
**Clinical stage**	
I	6 (7.8)
II	9 (11.7)
III	25 (32.5)
IVA	5 (6.5)
IVB	32 (41.5)
**≤Ⅱ**	15 (19.5)
**>Ⅱ**	62 (80.5)
**Treatment line**	
Neoadjuvant, adjuvant, or first-line therapy	43 (55.8)
≥ 2 lines of therapy	34 (44.2)
**Tumor involvement site (distant metastasis)**	
0	45 (58.4)
1	20 (26.0)
2	8 (10.4)
3	4 (5.2)
**Metastasis site**	
Lung	16 (20.8)
Liver	12 (15.6)
Brain	2 (2.6)
Bone	7 (9.1)
**Previous therapy**	
Chemotherapy	60 (77.9)
Radiotherapy	45 (58.4)
Surgery	36 (46.8)
**PD-L1 expression (CPS)**	
< 10%	14 (18.2)
≥ 10%	13 (16.9)
Unknown	50 (64.9)
**Immune checkpoint inhibitors**	
Sintilimab	18 (23.4)
Camrelizumab	42 (54.5)
Toripalimab	7 (9.1)
Tislelizumab	5 (6.5)
Pembrolizumab	5 (6.5)

CPS, combined positive score.

### Treatment outcome and potential predictors

**Figure 2:**
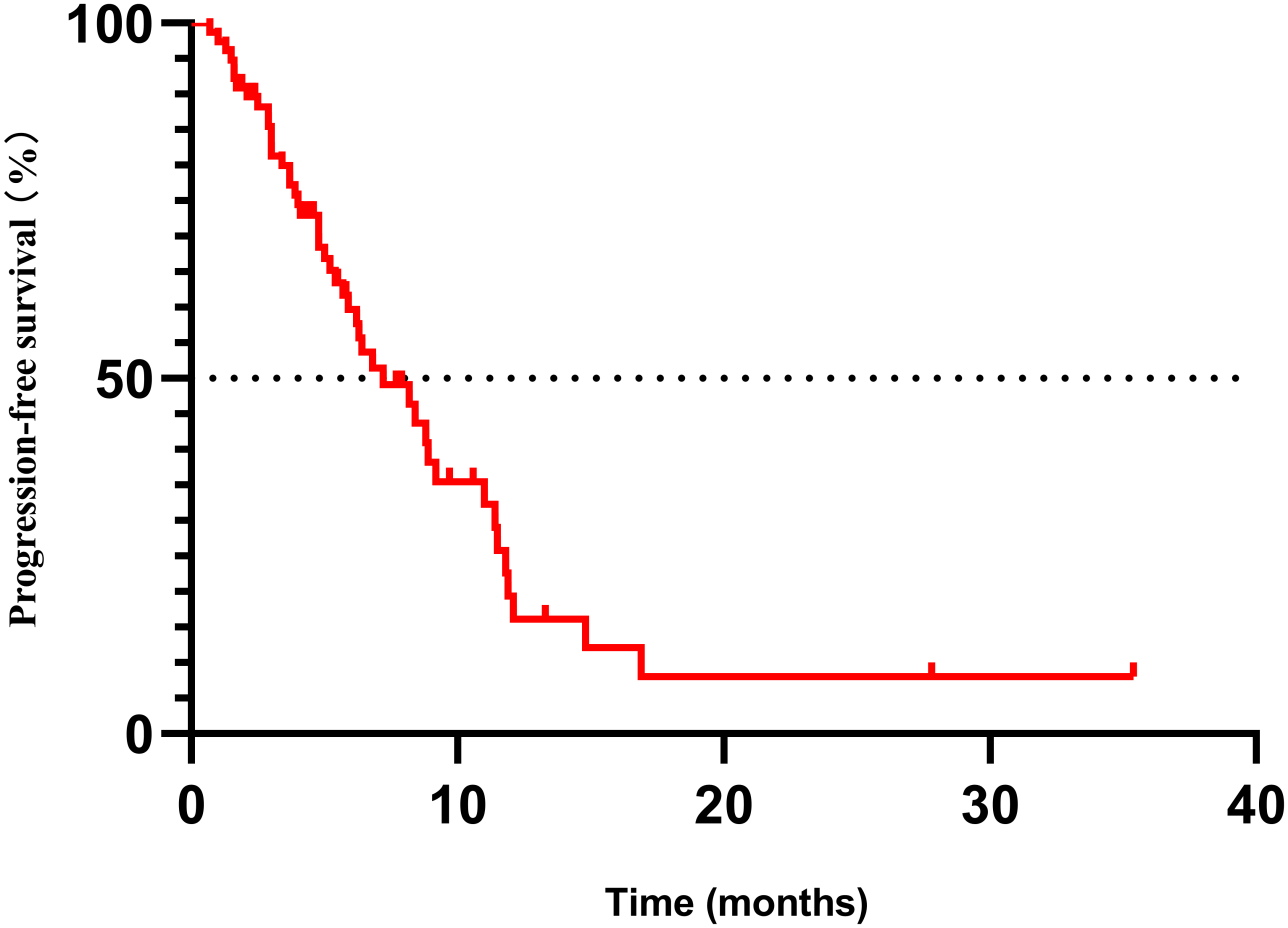
Kaplan–Meier plot for the total population (*n* = 77). PFS, progression-free survival.

The median PFS for all patients was 7.2 months (**Figure 2**). The median OS has not yet been reached as of the deadline.

**Figure 3:**
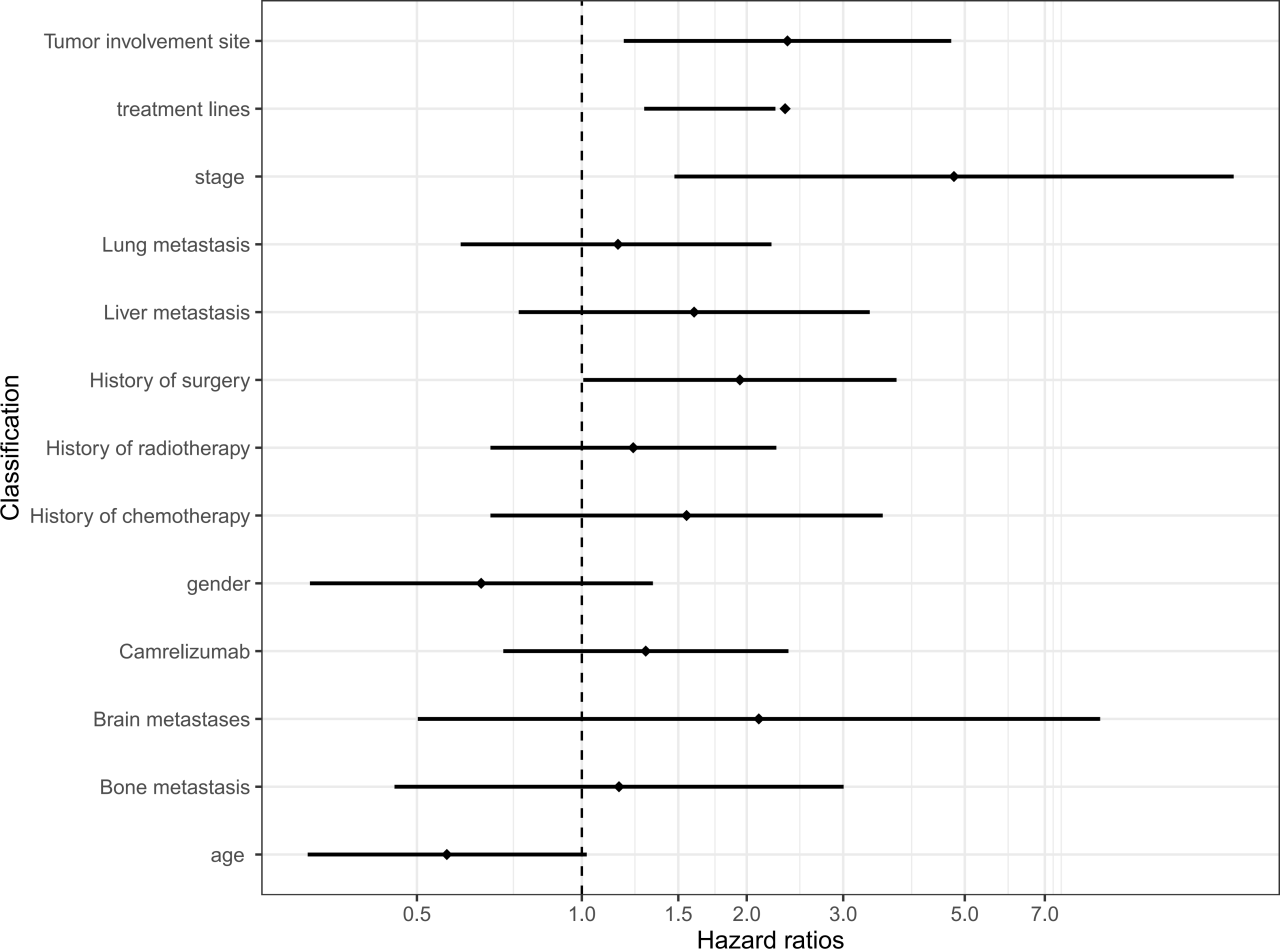
Univariate Cox regression models for progression-free survival. CI, confidence interval; HR, hazard ratio; Carrelizumab, Carrelizumab or others immune checkpoint inhibitors.

We conducted a univariate analysis of the potential influencing factors of PFS in patients using regression analysis of Cox proportional hazards. According to the results of the analysis, among all the clinical baseline characteristics included in the evaluation, the clinical-stage higher than stage II (HR = 4.778, 95% CI 1.476, 15.469), metastatic sites more than 2 (HR = 2.373, 95% CI 1.193, 4.719), treatment higher than first-line (HR = 2.350, 95% CI 1.300, 2.254), and previous surgical treatment (HR = 1.943, 95% CI 1.006, 3.750) were significantly related to the prognosis of patients. However, the age, gender, with/without lung, liver, brain, or bone metastases, whether the patients have received chemoradiotherapy, the type of ICI, and the expression of PD-L1 CPS was not significantly correlated with the patient’s PFS (*P*>0.05) (**Table 2**). The above conclusions are visually explained by plotting a forest map (**Figure 3**).

**Table 2:** Univariate and multivariate Cox regression models for progression-free survival.

Characteristics (Reference)	Univariate analysis		*P*-value	Multivariate analysis		*P*-value
	HR	95% CI		HR	95% CI	
Age (<60)	0.567	0.316-1.020	0.058			
Gender (male)	0.655	0.319-1.347	0.250			
Stage (≤II)	4.778	1.476-15.469	0.009	4.023	1.219-13.282	0.022
Tumor involvement site(≤2)	2.373	1.193-4.719	0.014	2.001	0.995-4.021	0.052
Treatment line (neoadjuvant,adjuvant, or first-line therapy)	2.350	1.300-2.254	0.005	2.016	1.096-3.708	0.024
Lung metastasis (none)	1.164	0.601-2.217	0.652			
Liver metastasis (none)	1.603	0.767-3.351	0.210			
Brain metastasis (none)	2.104	0.502-8.825	0.309			
Bone metastasis (none)	1.169	0.455-3.002	0.745			
History of chemotherapy (none)	1.553	0.681-3.539	0.295			
History of radiotherapy (none)	1.241	0.681-2.263	0.481			
History of surgery (none)	1.943	1.006-3.750	0.048	1.937	0.971-3.865	0.061
Carrelizumab or others (others)	1.308	0.719-2.381	0.379			

CI, confidence interval; HR, hazard ratio; Carrelizumab or others, Carrelizumab or others immune checkpoint inhibitors.

**Figure 4:**
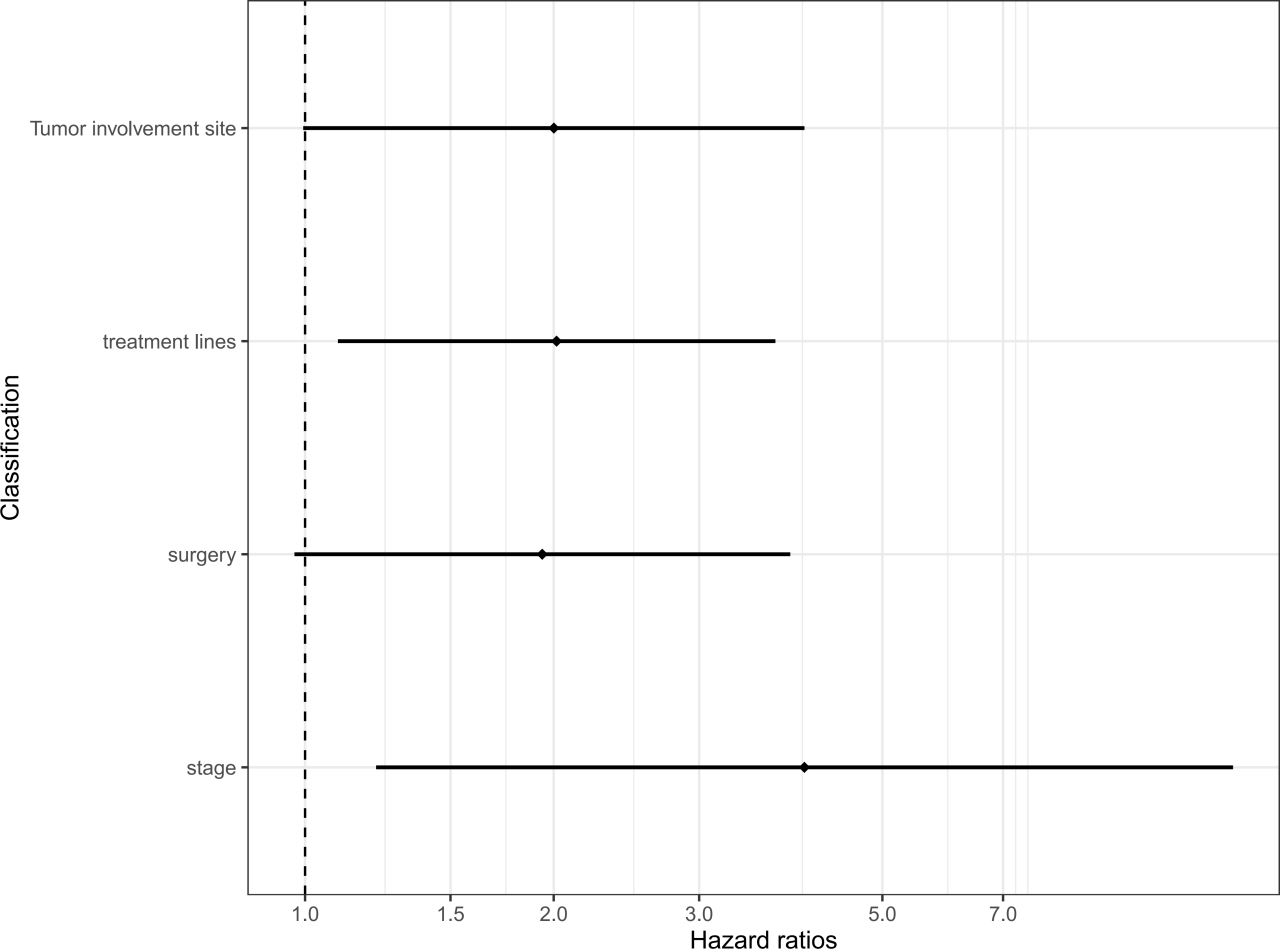
Multivariate Cox regression models for progression-free survival. CI, confidence interval; HR, hazard ratio.

Furthermore, we conducted further multivariate analysis on the four valuable clinical characteristics of the above univariate analysis, and the results showed that the clinical stage higher than stage II (HR = 4.023, 95% CI 1.219, 13.282), and treatment higher than first-line (HR = 2.016, 95% CI: 1.096, 3.708) were still an independent predictor of PFS (**Table 2**) (**Figure 4**).

### Subgroup analysis

**Figure 5:**
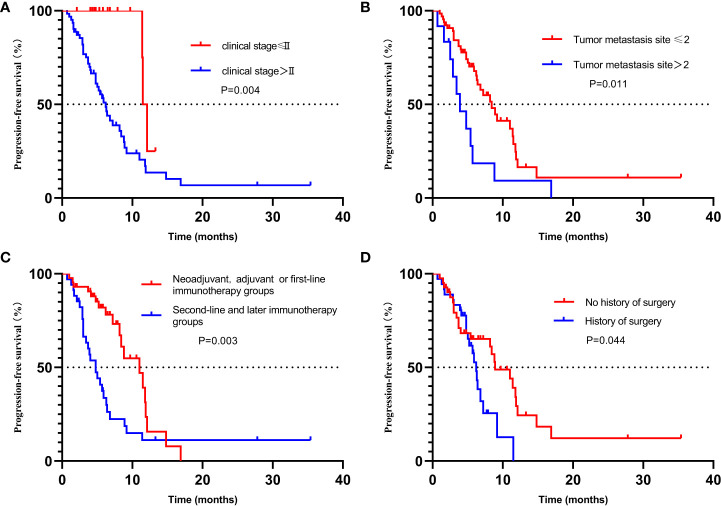
Kaplan–Meier plot for progression-free survival stratified by clinical factors, including **(A)** stage, **(B)** Number of metastatic lesions, **(C)** treatment lines, and **(D)** History of surgery. CI, confidence interval; PFS, progression-free survival.

The Kaplan–Meier method was used to analyze the potential influencing factors of patients with PFS, that is, further subgroup analysis on the clinical stage higher than stage II, the metastatic site more than 2, the treatment higher than the first line, and the previous surgical treatment. As shown in the figure, the median PFS of patients with clinical stage lower than or equal to stage II and higher than stage II was 11.5 months versus 6.2 months, *P* = 0.004 (**Figure 5A**). Patients with less than two sites of metastasis may have a longer PFS, with a median PFS of 8.4 months versus 3.9 months for those with more than two sites, *P* = 0.011 (**Figure 5B**). The median PFS was 11.0 months versus 4.8 months in neoadjuvant, adjuvant, or first-line immunotherapy groups versus second-line and later immunotherapy groups, *P* = 0.003 (**Figure 5C**). The median PFS of patients who had not received surgery (radical radiotherapy or chemotherapy or immunotherapy alone), and those who received surgery was 8.9 months versus 6.2 months, *P* = 0.044 (**Figure 5D**). This suggests that patients undergoing surgical treatment may have a worse prognosis than patients undergoing radical chemoradiotherapy.

In addition, we conducted a further subgroup analysis of 27 patients with recorded baseline PD-L1 expression. Of the 27 patients, 16 patients used carrelizumab, seven patients sintilizumab, and the rest patients teriprizumab (three cases), tislelizumab (one case), and pembrolizumab (one case). We found no significant statistical difference in the PFS comparison of each sub-group with PD-L1 CPS higher than 1, 5, and 10%, PD-L1 TPS higher than 1%, and whether PD-L1 is simultaneously expressed in tumor cells and immune cells as the sub-group grouping basis (**Table 3**).

**Table 3:** Subgroup analysis: Univariate Cox regression models for progression-free survival about PD-L1 expression level.

Characteristics	HR	95% CI	*P*-value
PD-L1 CPS≥ 1%	2.384	0.531-10.705	0.257
PD-L1 CPS≥ 5%	0.876	0.337-2.274	0.786
PD-L1 CPS≥ 10%	0.932	0.362-2.398	0.884
PD-L1 TPS≥ 1%	0.886	0.417-2.754	0.886
Simultaneous expression on tumor cells and immune cells	1.241	0.421-3.659	0.695

CPS, combined positive score; TPS, tumor proportion score.

We grouped patients with similar treatment strategies among the included patients and found that 46 of them were treated with immune-combination chemotherapy regimens. This included stages II (three patients), III (nine patients), IVA (two patients), IVB (20 patients) patients who received second or multiple lines of therapy after progression, and 4b patients (12 patients) who received first-line therapy. In this treatment subgroup, the median PFS for all patients was 5.9 months. Treatment lines ≥2 and metastatic sites >2 were independent risk factors for patients’ PFS. In contrast, the staging was no longer critical in affecting prognosis. Suggesting that early intervention remains the key to improving prognosis after the failure of first-line therapy or in immune combination therapy for advanced EPC (**Table 4**). The median PFS for patients who received neoadjuvant, adjuvant, or first-line therapy was 11.0 months. In contrast, the median PFS of immunotherapy patients after failure of multiple lines of therapy was 4.8 months, which had not been reached in the stage 2 patient group, 4.8, months in the stage 3 patient group, and 4.0 months in the stage 4 patients.

**Table 4:** Univariate and multifactorial analysis of PFS in the immune combination chemotherapy subgroup.

Characteristics (Reference)	Univariate analysis		*P*-value	Multivariate analysis		*P*-value
	HR	95% CI		HR	95% CI	
Age (<60)	0.554	0.276-1.109	0.095			
Gender (Male)	0.616	0.261-1.456	0.270			
Stage (≤II)	4.723	0.637-35.027	0.129			
Stage (≤III)	1.212	0.522-2.814	0.654			
Tumor involvement site(≤2)	2.124	1.021-4.415	0.044	2.784	1.277-6.069	0.010
Treatment line (neoadjuvant,adjuvant, or first-line therapy)	2.545	1.043-6.209	0.040	2.766	1.063-7.201	0.037
Lung metastasis (none)	0.987	0.485-2.009	0.971			
Liver metastasis (none)	1.296	0.598-2.810	0.511			
Brain metastasis (none)	1.707	0.401-7.262	0.469			
Bone metastasis (none)	1.045	0.400-2.726	0.929			
History of chemotherapy (none)	1.374	0.583-3.239	0.468			
History of radiotherapy (none)	1.196	0.579-2.473	0.628			
History of surgery (none)	2.237	1.034-4.839	0.041	1.943	0.875-4.341	0.103
Carrelizumab or others (others)	1.474	0.717-3.027	0.291			

### AE analysis

We mainly followed up the patients for thyroid function, bone marrow function, liver function, treatment-related esophagotracheal fistula, and AEs with digestive system diarrhea as the main symptoms and described the occurrence of the abovementioned related AEs during treatment with ICI. Among the 77 patients, 20 patients experienced AEs of varying degrees (25.97%). Of the 20 patients, five patients experienced grade 3/4 AE (6.49%), bone marrow suppression, liver damage (grade 4), diarrhea, and esophagotracheal fistula, respectively. The AE of highest incidence was 12 patients with myelosuppression (15.58%), followed by liver damage (alanine transpeptidase elevation) in six patients (7.79%), of which one patient experienced grade 4 liver functional impairment, and three patients had grade 1 liver damage, and two patients had grade 2 liver damage. Three patients had hypothyroidism (3.90%) (two of grade 1 and one of grade 2), and four patients had diarrhea (5.19%), grades 1, 2, and 3 each. Another patient developed esophagotracheal fistula during treatment. Four patients experienced more than two of the above AEs, and one patient experienced three AEs of liver insufficiency, leukopenia, and diarrhea at the same time (**Table 5**).

**Table 5:** Treatment-related adverse events according to category and grade.

Adverse events	Grand				
	1	2	3	4	5
Hypothyroidism	2	1	0	0	0
Myelosuppression	3	7	1	1	0
Abnormal liver function	3	2	0	1	0
diarrhea	1	2	1	0	0
A patient has an esophagotracheal fistula.					

## Discussion

ICIs have become one important strategy for the first- and second-line treatment of EPC. In previous clinical studies, ICIs treatment has shown good efficacy and safety in the treatment of EPC. Our retrospective study aims to study the efficacy and safety of immunotherapy for patients with EPC in the real world. The median PFS in our trial was 7.2 months, with a median mPFS of 11.0 months in patients who received neoadjuvant, adjuvant immunotherapy, or first-line immunotherapy, which was greater than in prior studies (11–13). The early stages and the good physical condition may be the key to a better mPFS. Furthermore, the kind of ICI medication, changes in baseline PDL-1 expression in patients, and disparities in the races included in the study should all be taken into account (14).

Existing studies have proven the efficacy of immunotherapy for EPC, but not all patients can benefit from immunotherapy, which mainly depends on the tumor microenvironment (9). In other words, a suitable biomarker can help us further screen out potential benefit groups for immunotherapy for EPC. Among the common immunotherapy biomarkers, the expression of PD-L1 has been most extensively studied (15). In a meta-analysis involving 4,174 patients with advanced tumors (including lung cancer, kidney cancer, head and neck cancer, melanoma, and urothelial cancer), the patients received nivolumab, pembrolizumab, or atezolizumab treatment, respectively, and the analysis results showed that both PD-L1-positive and PD-L1-negative patients can benefit from PD-1 or PD-L1 blocking therapy, in which the survival benefit of pembrolizumab treatment for PD-L1-negative patients was minimal. In all selected subgroups, PD-1 or PD-L1 inhibitors are more effective in PD-L1-positive patients than in PD-L1-negative patients (16). According to the results of PD-L1 stratified analysis of KEYNOTE-189 (17) and KEYNOTE-407 studies (18), pembrolizumab combined with platinum-containing chemotherapy can bring OS and PFS benefits regardless of the expression status of PD-L1. In the KEYNOTE-180 trial, patients with high PD-L1 expression had a higher 1-year OS than patients with low PD-L1 expression (35% vs. 22%) (19). Quite a lot of existing studies have shown that patients with higher PD-L1 expression seem to get more benefits from ICI treatment. In this study, there was no significant statistical difference between PD-L1 high-expression and low-expression groups. We still found no significant correlation with prognosis after changing the definition of PD-L1 high expression (PD-L1 CPS ≥ 1%, 5%, 10%, CPS is defined as the sum of PD-L1-positive tumor cells, macrophages, and lymphocytes divided by total tumor cells). This may be related to insufficiencies of patients with recorded PD-L1 expression included in the statistics and the resulting bias. The difference in the efficacy of different types of ICI on PD-1 expression also affected our interpretation of the final result. Due to limited data from current clinical studies, the correlation between PD-L1 status and clinical results should be further verified. In addition, due to lack of data, we had not conducted further analysis and comparison of other potential biomarkers for EPC, such as MSI, PD-L2, TMB, and so forth (20) in this study.

According to a systematic review published by Sjoerd M. L et al., age was not related to the prognosis of EPC, and most studies do not support gender as a prognostic factor (21). Similar studies by Gregory O’Grady and MARKER S et al. also believed that age was not related to the prognosis of EPC (22, 23). A study by Pierre Bohanes et al. also believed that the female had a better prognosis than the male (24). A study by Yutong He et al. also believed that the female has a better prognosis than the male (25). In another retrospective study by Jiaxin Li et al. on prognosis analysis of non-surgical early stage EPC chemoradiotherapy, in a total of 3,736 patients included, multivariate Cox regression analysis showed that the age, gender, treatment, and cause of surgery are independent predictors of OS (26). In our study, we did not find a significant correlation between the age and gender of patients with EPC with the PFS of the patients in immunotherapy. However, because we did not pay attention to the correlation between OS with the age and gender of patients, further follow-up observation should be conducted on the patients included in the study.

The review by Véronique Vendrely et al. mentioned that the T stage of the tumor was a factor affecting the prognosis of EPC. There are different 5-year survival rates according to different T stage of tumor: 74% for ypT0 lesions, 83% for pTis lesions, 67% for pT1 lesions, 49% for pT2 lesions, and 30% for pT3 lesions (27). The study by Sjoerd M.L et al. believed that lymph node metastasis (N stage) may be a poor prognostic factor for EPC (21). It was reported that the 5-year survival rate for pN0 lesions was 63% and that for pN + lesions was 30% (28). In an analysis of survival of patients after EPC resection by Feng Du et al., a statistical analysis was performed on 4,566 eligible patients in the SEER database. The results showed that AJCC T stage, AJCC N stage, and chemoradiotherapy were independent influencing factors of EPC survival (29). While, in the analysis of the clinical characteristics of 5,283 cases of EPC by Yutong He et al., it was found that pathological stage was an independent predictor of patient prognosis (stage II: HR = 1.80, 95% CI: 1.40, 2.31; stage III: HR = 2.62, 95% CI: 2.06, 3.34; stage IV: HR = 3.90, 95% CI: 2.98, 5.09). Because the patients we included in the study were mainly stages III and VI, we conducted a combined analysis of the stages of the included patients and finally found that patients with clinical stages higher than stage II had worse PFS than patients with stages I and II (11.5 months vs. 6.2 months, *P* = 0.004), and this conclusion was confirmed again by multivariate analysis (HR = 4.778, 95% CI: 1.476, 15.469). Also, due to the limitation of the sample size of grouping, we had not conducted further analysis on the subgroups of non-surgical patients and surgical patients of different stages. In addition, we did not find a significant difference in PFS in different subgroups of the T stage and N stage during the study process. Because there have been no sample studies on prognostic factors of EPC immunotherapy, this conclusion still should be verified by subsequent further studies.

In a study of immunotherapy for lung cancer by Junlin Yao et al., it was found that liver metastasis (HR = 3.7; 95% CI: 1.6, 8.5; *P* < 0.01) and ≥ 3 line therapy (HR = 3.5; 95% CI: 1.7, 7.4, *P* < 0.01) was a poor predictor of PFS (30). In our statistical process of patients with lung, liver, brain, and bone metastases from EPC in this study, we did not find a significant correlation with the PFS of immunotherapy. In the univariate analysis, we found that patients with less than or equal to two metastasis sites had longer mPFS (8.4 months vs. 3.9 months, *P* = 0.011), but in multivariate analysis, there was no significant difference between the two groups (HR = 2.001; 95% CI: 0.995. 4.021, *P* = 0.052), which may be due to the limited sample size of distant metastases included, so a larger study cohort is needed to better clarify the conclusion. Notably, for the treatment subgroup of immune combination chemotherapy, both univariate and multivariate analyses suggested that the number of metastatic sites was associated with prognosis.

Although we have seen a potential in first-line immunotherapy for EPC from the published interim data of existing phase III clinical studies (14), we still expect the release of final experimental data and further stratified analysis of the treatment lines to further clarify the best timing for immunotherapy intervention. Our study compared the PFS of immunotherapy in different treatment lines of EPC patients and finally found that the prognosis of the first-line immunotherapy patient subgroup was better than that of the latter-line treatment subgroups (11.0 months vs. 4.8 months *P* = 0.003).

In the process of univariate analysis, we found that patients in the group that received surgical treatment previously had worse PFS than those in the group that had not received surgical treatment (received or not received radical chemoradiotherapy), which seems to be different from our previous understanding. Although multivariate analysis ultimately rejected the decisive role of surgical treatment on PFS, what still cannot be ignored is that, for patients with inoperable EPC, immunotherapy and its combination with chemoradiotherapy or anti-vascular–targeted therapy, especially mechanisms of chemotherapy activating tumor immunogenicity and remote effect and the synergistic effect of immunotherapy (31) have long-term prognostic benefits for patients with EPC.

In the treatment subgroup of immunotherapy combined with chemotherapy, the median PFS in patients treated with second-line and multiple lines was 4.8 months, a figure superior to the previously reported 2.5 months (32, 33), thanks to the small number of stage 2 and three patients included in this subgroup. According to Matsubara, Y et al. (34), for the second-line treatment of advanced EPC, the median PFS for CPS ≥ 10 was 4.1 months. We obtained similar results (mPFS = 4.0 months) for patients treated in the advanced second line, although we did not obtain complete PD-L1 expression data. Of course, the difference in the type of immunosuppression remains a potential influence on this outcome. As for stage 2 and three patients, according to Ronan J et al. (4), the median DFS of Nivolumab maintenance therapy was 22.4 months in patients with stage 2 or 3 EPC after surgery after the neoadjuvant radiotherapy. We performed immunotherapy in combination with chemotherapy in patients with stage 3 EPC after a failure of multiple lines of therapy. The median age of this group was 64 years, and the median PFS was 4.8 months. Sample size and the number of lines treated may be the main factors for the poor prognosis of this subgroup of patients. In addition, the overall high age is a factor that should be taken into account. Due to the lack of appropriate genetic testing, we were unable to assess patient resistance to immunotherapy or combination chemotherapy regimens after multiple lines of therapy. In addition, limited by the actual treatment situation in the real world, the included patients were not all examined comprehensively and systematically to exclude actual comorbid conditions.

Because almost all patients that we included were esophageal squamous cell carcinoma, and patients with tumors at the gastroesophageal junction were not included, further analysis of the pathological type and tumor location was not made. In addition, if our research group needs to follow up on the patients for PFS, a longer follow-up period and large sample size are needed to further study relevant data affecting the OS of patients.

In our study, we also evaluated the safety of immunotherapy for EPC. We mainly observed AEs such as thyroid function, bone marrow function, liver function, treatment-related esophagotracheal fistula, and AEs with digestive diarrhea as main symptoms, and immune-related cardiotoxicity was not noted during treatment. We noted that 20 patients who experienced the abovementioned recorded main AEs of varying degrees. Four patients experienced more than two immunotherapy-related AEs, in which one patient experienced three AEs of liver insufficiency, leukopenia, and diarrhea at the same time, but all were grade 1–2 events. Among all observed patients, the incidence of grades 3 and 4 AEs was 6.49%, which were bone marrow suppression, liver damage, diarrhea, and esophagotracheal fistula. Bone marrow suppression was the most frequent AE in this study, with an incidence of 15.58%. The second most frequent AE was liver insufficiency, with an incidence of 7.79%, in which one patient had liver damage of grade 4. The prevalence of hypothyroidism and diarrhea was 3.90 and 5.19%, respectively. Another patient developed esophagotracheal fistula during treatment. The main adverse reactions observed were roughly similar to those in previous reports (35, 36). The difference in the incidence of adverse reactions was mainly due to the fact that the immunotherapy regimen that we included in the study covered five different ICIs, and due to the limitation of observation time, we have limited records of treatment-related AEs, and some patients may have new or more serious adverse reactions in follow-up treatment. The limit of sample size may also affect the results of the study. In addition, the mixed toxicity caused by combination therapy such as chemoradiotherapy cannot be completely ruled out in about 60% of AEs. One patient with esophagotracheal fistula discontinued the drugs and was given palliative treatment. The other patients with grades 3 and 4 responses were treated with methylprednisolone 1–2 mg/kg after drug withdrawal, and the AE symptoms were all relieved significantly.

In the real world, the strategy selected for patients should not only follow guidance but also consider cost, drug availability, and the patient’s willingness. Because of the need for treatment, patients with hypertension, diabetes, coronary heart disease, chronic viral hepatitis (non-replicating period), chronic pneumonia, hyperthyroidism, chronic renal insufficiency, and so forth are not excluded. Corresponding treatment drugs for them such as effects of drugs affecting intestinal flora on immunotherapy cannot be completely excluded from treatment.

## Conclusions

In summary, we clarified the efficacy and safety of ICI treatment of EPC in the real world. In addition, a preliminary study has been conducted on potential independent predictors of PFS, and preliminary exploration has been made on the timing of ICI intervention in patients with EPC and the applicable population. However, the above conclusions still need to be further confirmed by larger scale prospective studies in the future.

## Data availability statement

The raw data supporting the conclusions of this article will be made available by the authors, without undue reservation.

## Ethics statement

The studies involving human participants were reviewed and approved by the ethical review board committee of 900th Hospital of the Joint Logistics Team. The patients/participants provided their written informed consent to participate in this study.

## Author contributions

Conception and design: ZF and HL. Administrative support: ZF. Provision of study materials: XW, MW, LC, GL, YZ, XY, PM, and XL. Collection and assembly of data: ZF, XW, MW, LC, and GL. Data analysis and interpretation: HL, YZ, XY, PM, and XL. Manuscript writing: all authors. All authors contributed to the article and approved the submitted version.

## Funding

This study was supported by Natural Science Foundation of Fujian Province (grant number: 2021J011259).

## Acknowledgments

The authors thank Zhu Z.A. and Ma L. for the collection of data.

## Conflict of interest

The authors declare that the research was conducted in the absence of any commercial or financial relationships that could be construed as a potential conflict of interest.

## Publisher’s note

All claims expressed in this article are solely those of the authors and do not necessarily represent those of their affiliated organizations, or those of the publisher, the editors and the reviewers. Any product that may be evaluated in this article, or claim that may be made by its manufacturer, is not guaranteed or endorsed by the publisher.
